# Root and nitrate-N distribution and optimization of N input in winter wheat

**DOI:** 10.1038/s41598-019-54641-w

**Published:** 2019-11-29

**Authors:** Bin-Bin Guo, Bei-Cheng Liu, Li He, Yang-Yang Wang, Wei Feng, Yun-Ji Zhu, Nian-Yuan Jiao, Chen-Yang Wang, Tian-Cai Guo

**Affiliations:** 1 National Engineering Research Centre for Wheat, State Key Laboratory of Wheat and Maize Crop Science, Henan Agricultural University, #15 Longzihu College District, Zhengzhou, Henan 450046 P.R. China; 2 College of Agronomy, Henan University of Science and Technology, Luoyang, 471023 P.R. China

**Keywords:** Plant ecology, Plant physiology

## Abstract

Scientific management of nitrogen (N) fertilizer has a significant effect on yield while also reducing the environmental risks. In this study, we conducted field experiments over three years at two different sites (Zhengzhou and Shangshui) in Henan Province, China, using different N application rates (0, 90,180, 270, and 360 kg ha^−1^) to determine the relationships between soil N supply and N demand in winter wheat (*Triticum aestivum* L.). Optimal N input was then determined. Both sites showed the same trend. Namely, aboveground N uptake and soil nitrate N (NO_3_^−^-N) increased with increasing N, while NO_3_^−^-N decreased with increasing soil depth, gradually moving downwards with growth. A significant correlation (*p* < 0.001) between increasing aboveground N uptake and increasing NO_3_^−^-N was also observed under N application, with the best relationships occurring in the 20–60 cm layer during jointing-anthesis (*R*^2^ = 0.402–0.431) and the 20–80 cm layer at maturity (*R*^2^ = 0.474). Root weight density showed the same spatial-temporal characteristics as NO_3_^−^-N, following a unimodal trend with increasing N, and peaking at 90 kg ha^−1^. The root weight density was mainly distributed in the 0–60 cm layer (above 80%), with the 20–60 cm layer accounting for 30% of the total root system. In this layer, the root weight density was also significantly positively correlated with aboveground N uptake. Wheat yield reached saturation under high N (>270 kg ha^−1^), with a sharp decrease in N use efficiency (NUE) and linear increase in residual NO_3_^−^-N. To balance yield and the risk of environmental pollution in the experimental area, an N application rate of 180–270 kg ha^−1^ is recommended under sufficient irrigation, thereby supporting a well-developed root system while ensuring balance between N supply and demand.

## Introduction

Wheat is a major food crop worldwide, with a cultivated area of 2.18 × 10^8^ ha and accounting for more than 20% of global arable land^1^. Henan Province is the main wheat producing area in China, representing 22.1% of the entire production area and producing 25% of the national wheat output. N fertilizer is a crucial nutrient for wheat growth and grain yield formation. However, understanding the crop N demand at different growth stages is challenging and, as a result, the input rate of chemical N fertilizer tends to surpass crop needs^2^. A large amount of N fertilizer therefore remains in the soil profile, reducing the nitrogen use efficieny (NUE), increaisng the environmental risk and reducing economic return^3,4^.

Under increasing land use and intensive crop production, rainfed and irrigated agriculture are unsustainable without fertilization; thus, N fertilizers are extensively applied, leading to an increase in N consumption worldwide. Meanwhile, soil nitrate N (NO_3_^−^-N) build-up in the soil profile is also attracting increasing attention. For example, Ju *et al*.^5^ found that a large amount of NO_3_^−^-N accumulates in the soil profile, mainly due to inblanace between high N fertilizer input and crop N needs for maximum yield, and high summer rainfall. Similarly, Lenka *et al*.^6^ found that the NO_3_^−^-N content in the soil profile was higher under an N application rate of 180 kg ha^−1^ throughout maize-wheat cropping in a semi-arid region. Zheng *et al*.^7^ also demonstrated NO_3_^−^-N leaching from flowering to maturity in winter wheat under plowing tillage and subsoiling treatment, with a subsequent increase in accumulation of NO_3_^−^-N in the 100–160 cm soil layer. Optimization of N fertilizer management and a reduction in the waste of resources is therefore a key issue in wheat production.

Crop growth therefore needs to match N fertilizer in the soil, while ensuring a low risk of environmental pollution. In the past decade, a number of studies have detailed the dynamic changes in NO_3_^−^-N at different growth stages and in different soil layers. For example, Feng *et al*.^8^ found that NO_3_^−^-N mainly accumulated in the 60–80 cm layer with bed planting and the 80–100 cm layer with flat planting. Furthermore, Xia *et al*.^9^ demonstrated that the NO_3_^−^-N content in the 0–60 cm soil layer was highest at the beginning of winter, subsequently decreasing with growth. A high soil NO_3_^−^-N content was also found in the topsoil layer 36 days after sowing maize on the Loess Plateau^10^. Meanwhile, the relationship between NO_3_^−^-N and physiological indices was previously reported by Miao *et al*.^11^, who revealed that initial NO_3_^−^-N accumulation in the 0–100 cm soil layer at maturity had the highest correlation with wheat biomass. Moreover, Cui *et al*.^12^ demonstrated a ‘linear plus plateau’ relationship between summer maize biomass at the ten-leaf stage and the soil NO_3_^−^-N content in the 0–90 cm layer. Maize grain yield and N uptake responses to pre-planting soil NO_3_^−^-N content were also found to simulate a ‘linear plus plateau’ model across plots^13^. However, few studies have systematically investigated the relationship between soil NO_3_^−^-N content and aboveground N uptake in winter wheat across widely varying field environments. A detailed understanding of the dynamic relationship between crop N demand and soil N supply could therefore provide a theoretical foundation for the optimization of N fertilizer application in winter wheat.

In this paper, based on experiments carried out over three years at two sites in Henan Province, we analyzed wheat aboveground N uptake, grain yield, NUE, root distribution, and the NO_3_^−^-N content in the 0–100 cm soil layer. The objectives were to: (1) determine the spatial and temporal dynamics of the root system and soil NO_3_^−^-N; (2) clarify the relationship between soil N supply and wheat N demand; and (3) recommend an optimal N rate for improving grain yield and NUE in winter wheat, while reducing the waste of resources. The results provide a theoretical basis for fertilization strategies in winter wheat production.

## Results

### Effects of the experimental factors on wheat yield, NUE and soil NO_3_^−^-N

The effect of year (Y), site (S) and N rate (N) on wheat yield and NUE is shown in Table 1. The effect of N on yield was extremely significant (*p* < 0.001), while the interaction effects of S-N and Y-S-N had remarkable effects (0.001 < *p* < 0.05). The effects of Y and N on NUE were also extremely significant, although the interaction effects of these three factors was not. As shown in Table 2, these four factors and most of the interaction effects were significant in terms of soil NO_3_^−^-N, highlighting the importance of the environmental conditions. Table 3 showed that the yield increased as the N rate increased from 0 to 270 kg ha^−1^ but decreased at 360 kg ha^−1^. The difference between N270 and N360 is not significant, but significantly higher than N0, N90 and N180. The content of soil NO_3_^−^-N increased with the increase of N rate at different growth stages. N180, N270 and N360 is significantly higher than N0 and N90 (Table 4).

**Table 1:** The effect of year, site and N rate on wheat yield and NUE.

Source of variation	Yield (kg ha^−1^)	NUE (%)
Year(Y)	0.46	14.23**
Site(S)	2.13	0.02
N rate(N)	193.87**	284.06**
Y*N	1.31	2.30
S*N	3.54*	4.98
Y*S*N	2.96*	1.81

**, and *, indicate that the *F* of the effect is significant for *p* < 0.001 and 0.001 < *p* < 0.05, respectively.

**Table 2:** The effect of year, site, N rate and soil layer on soil NO_3_^−^-N.

Source of variation	Soil NO_3_^−^-N (kg ha^−1^)
Year(Y)	143.04**
Site(S)	172.91**
N rate(N)	41.19**
Soil layer(L)	100.52**
Y*N	8.92**
Y*L	6.42**
S*N	15.02**
S*L	8.17**
Y*S*N	12.91**
Y*S*L	7.89**
Y*N*L	1.98*
S*N*L	0.73
Y*S*N*L	3.01**

**, and *, indicate that the *F* of the effect is significant for *p* < 0.001 and 0.001 < *p* < 0.05, respectively.

**Table 3:** Effects of N rate on yield under different years and sites.

N rate	N	Zhengzhou			Shangshui	
		2013–2014	2014–2015	2015–2016	2014–2015	2015–2016
N0	3	4008.3d	3831.9c	4184.7d	3991.3c	3357.0d
N90	3	6326.8c	6112.9b	6540.6c	5596.7b	5090.1c
N180	3	7214.5b	7644.0a	7582.7b	6793.1b	7711.8b
N270	3	8256.4a	7980.2a	8532.5a	8479.0a	8372.3a
N360	3	8182.6a	7612.9a	7822.5ab	8057.4a	7712.9b

The small letters indicate significant difference at P < 0.05.

**Table 4:** Effects of N rate on soil NO_3_^−^-N at different growth stages.

N rate	N	Wintering	Reviving	Jointing	Booting	Anthesis	Maturity
N0	3	39.9d	25.4c	10.7c	11.0d	8.3c	14.3b
N90	3	54.8c	41.8b	22.0c	24.4cd	20.8bc	21.6ab
N180	3	63.2bc	44.3b	25.8bc	38.2bc	21.4bc	31.3a
N270	3	69.3ab	48.7ab	39.5ab	47.6ab	28.2b	28.3ab
N360	3	74.2a	52.7a	43.3a	56.1a	47.6a	34.8a

The dataset is from Zhengzhou in 2013–2014. The small letters indicate significant difference at P < 0.05.

### Dynamic changes in aboveground N uptake and soil NO_3_^−^-N at different growth stages

**Figure 1:**
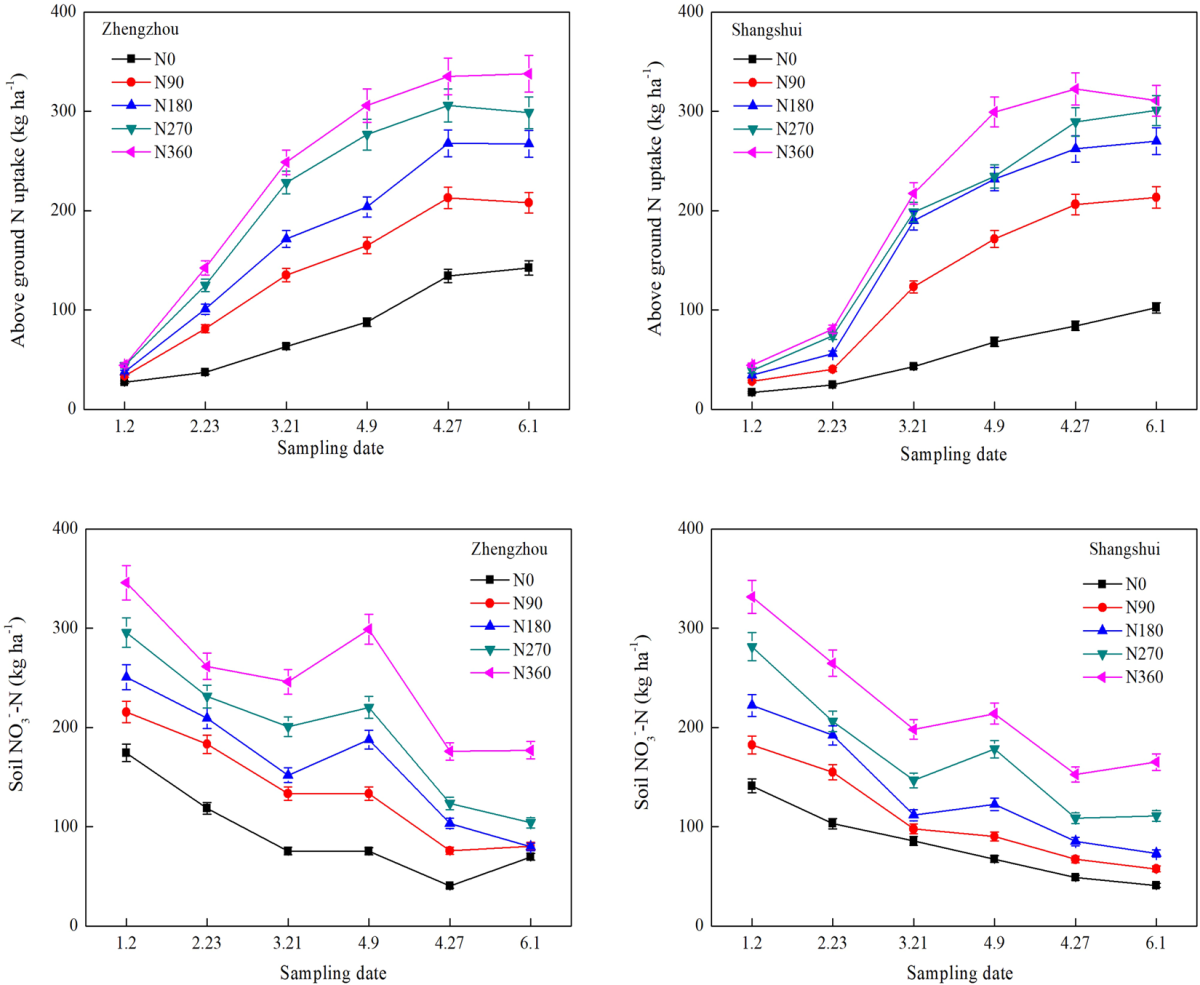
Dynamic changes in aboveground N uptake and NO_3_^−^-N in the 0–100 cm soil profile at different growth stages in Zhengzhou and Shangshui. 1.2, 2.23, 3.21, 4.9, 4.27 and 6.1 represent Wintering, Reviving, Jointing, Booting, Anthesis and Maturity growth stage, respectively.

Using the datasets from Zhengzhou and Shangshui in 2014–2015 as an example, Fig. 1 shows the dynamic changes of aboveground N uptake and NO_3_^−^-N in the 0–100 cm soil layer. Aboveground N uptake and soil NO_3_^−^-N in Zhengzhou and Shangshui ranged from 27–370 and 17–323 kg ha^−1^ and 40.3–345.8 and 41.0–331.4 kg ha^−1^, respectively. Under all N treatments, aboveground N uptake and soil NO_3_^−^-N increased with increasing N rate, and were significantly higher than under N0 treatment, manifesting in the trend of N360 > N270 > N180 > N90 > N0. Aboveground N uptake also increased gradually with growth stage. In contrast, NO_3_^−^-N gradually decreased with growth, with no significant differences between the two sites. At the wintering stage, NO_3_^−^-N remained high due to the high input of N fertilizer, under-development of the root system and low uptake of N. It then decreased from the reviving to jointing before rising again at booting following topdressing at the jointing stage. At anthesis-maturity, soil NO_3_^−^-N decreased due to vigorous growth.

### Root distribution in different soil layers under each N fertilizer rate

**Figure 2:**
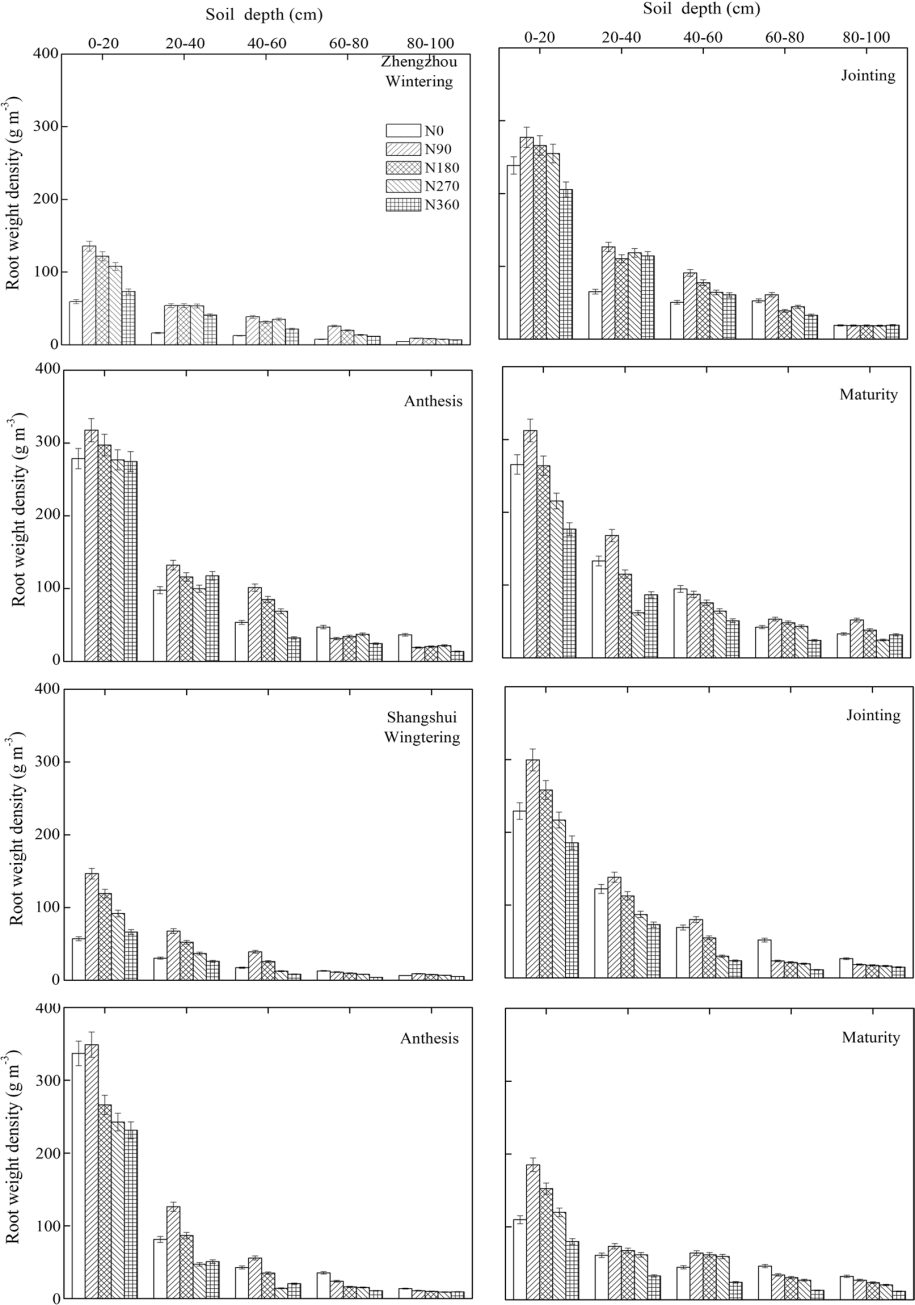
Effects of N rate on root distribution in different soil layers at different growth stages.

Root weight density was significantly affected by the N application rate and soil depth. As shown in Fig. 2, the root weight density decreased under all treatments with increasing soil layer, with significant differences between soil depths in both Zhengzhou and Shangshui. In both sites, the root dry weight presented the same trend, first increasing then decreasing with growth, with a peak at anthesis. The largest root weight density was observed under N90 treatment, after which it decreased with increasing N rate. The effect of N rate on root dry weight differed between different soil layers. In general, N rate had the most significant effect on middle and lower layers (20–60 cm), with high N rates (N270 and N360) inhibiting root growth (25–55%) compared to N90 treatment. The root system was mainly distributed in the 0–60 cm layer where the root weight density accounted for more than 80% of the total root system at different growth strages. The root weight density in the 20–60 cm layer represented approiximately 30% of the total root system. Under N0, the root weight density was lower than under N90 treatment in the 0–60 cm; however, an opposite trend was observed in the deep soil layer (60–100 cm) due to insufficient nutrients and subsequent downward growth of the roots.

### Distribution of NO_3_^−^-N in the soil profile under different N fertilizer rates

**Figure 3:**
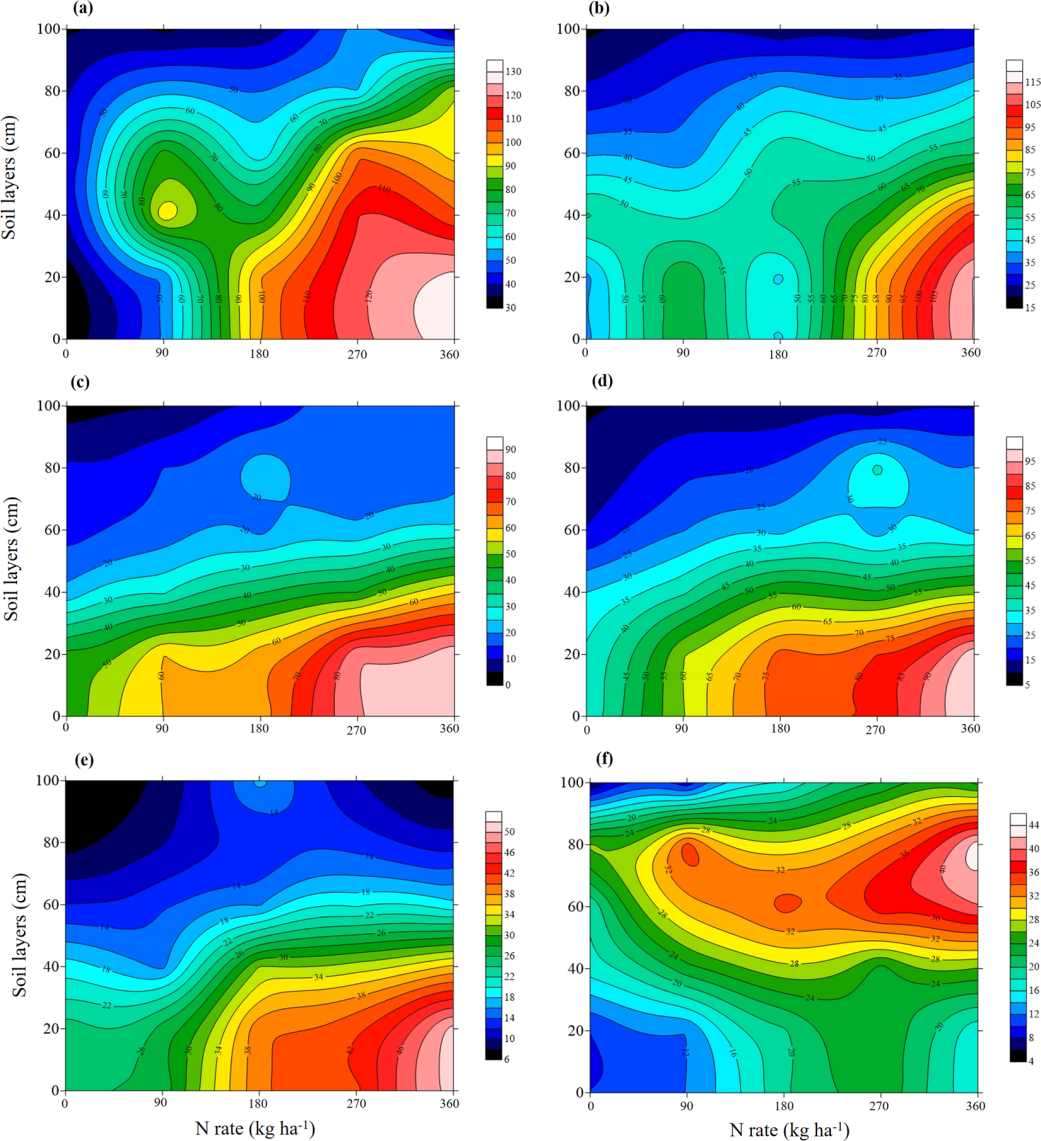
Effects of N fertilization rate on the NO_3_^−^-N (kg ha^−1^) in different soil layers at different growth stage. (**a–f**) represent datasets at wintering (**a**), reviving (**b**), jointing (**c**), booting (**d**), anthesis (**e**) and maturity (**f**).

The results from Zhengzhou in 2014–2015 are summarized in Fig. 3, showing the general distribution of NO_3_^−^-N under different N rates at different growth stages. Overall, total NO_3_^−^-N in the 0–100 cm soil profile increased with increasing N rate; however, this was not true for all soil layers. Moreover, the N treatment rate resulting in maximum NO_3_^−^-N also varied according to soil layer under different growth stages, with most occurring under 360 kg ha^−1^, some under 270 kg ha^−1^ and a few under 180 kg ha^−1^. High-value areas of NO_3_^−^-N always occurred at 270–360 kg ha^−1^, and varied with soil layer and growth stage. At wintering-reviving, high-value areas were within the 0–50 cm layer, while at jointing-anthesis they were found in the 0–30 cm layer, and at maturity in the 60–80 cam layer (Fig. 3). NO_3_^−^-N content decreased with increasing soil layer before anthesis after which values increased then decreased. NO_3_^−^-N moved down gradually with growth, resulting in a high NO_3_^−^-N concentration in the upper soil layer (0–40 cm) before anthesis and in the middle-lower soil (60–80 cm) after anthesis.

### Relationship between aboveground N uptake, NO_3_^−^-N and root weight density

The correlations between aboveground N uptake and root weight density were determined using data from Zhenghou and Shanghui in 2014–2015 (Table 5). The results showed a significant positive correlation between aboveground N uptake and root weight density in the 0–40 cm soil layer at wintering and in the 20–40 cm layer during jointing-anthesis. In contrast, significant negative correlations were observed in the 80–100 cm soil layer at jointing, the 60–100 cm layer at anthesis and the 40–80 cm layer at maturity. Overall, the root weight density in the upper and middle soil layers was positively correlated with aboveground N uptake, which is beneficial for absorption of soil N. Meanwhile, negative correlations were observed in middle and lower soil layers. Aboveground N uptake was positively correlated with NO_3_^−^-N in most soil layers during reviving-maturity, except for the 60–100 cm layer at reviving and the 80–100 cm layer at both jointing and anthesis. The correlation was poor in all soil layers at wintering. Although most correlation coefficients were significant, values were relatively low (*r* < 0.6), suggesting that further studies are needed to fully understand the relationship between soil N supply and wheat N demand.

**Table 5:** Correlation between aboveground N uptake, NO_3_^−^-N content and root weight density at different growth stages. *Represents a significant difference at 5%.

	Root weight density (*n* = 20)				NO_3_^−^-N content (*n* = 75)					
	Wintering	Jointing	Anthesis	Maturity	Wintering	Reviving	Jointing	Booting	Anthesis	Maturity
0–20 cm	0.439*	−0.138	−0.289	−0.196	−0.137	0.390*	0.528*	0.592*	0.340*	0.322*
20–40 cm	0.489*	0.565*	0.552*	−0.319	0.083	0.269*	0.558*	0.544*	0.454*	0.442*
40–60 cm	0.329	0.372	0.045	−0.473*	0.060	0.276*	0.477*	0.341*	0.315*	0.468*
60–80 cm	0.382	−0.282	−0.556*	−0.449*	0.101	0.217	0.495*	0.379*	0.262*	0.507*
80–100 cm	0.212	−0.582*	−0.475*	−0.216	0.023	0.190	0.224	0.308*	0.125	0.417*

### Relationship between increased aboveground N uptake and NO_3_^−^-N in different soil layers

**Figure 4:**
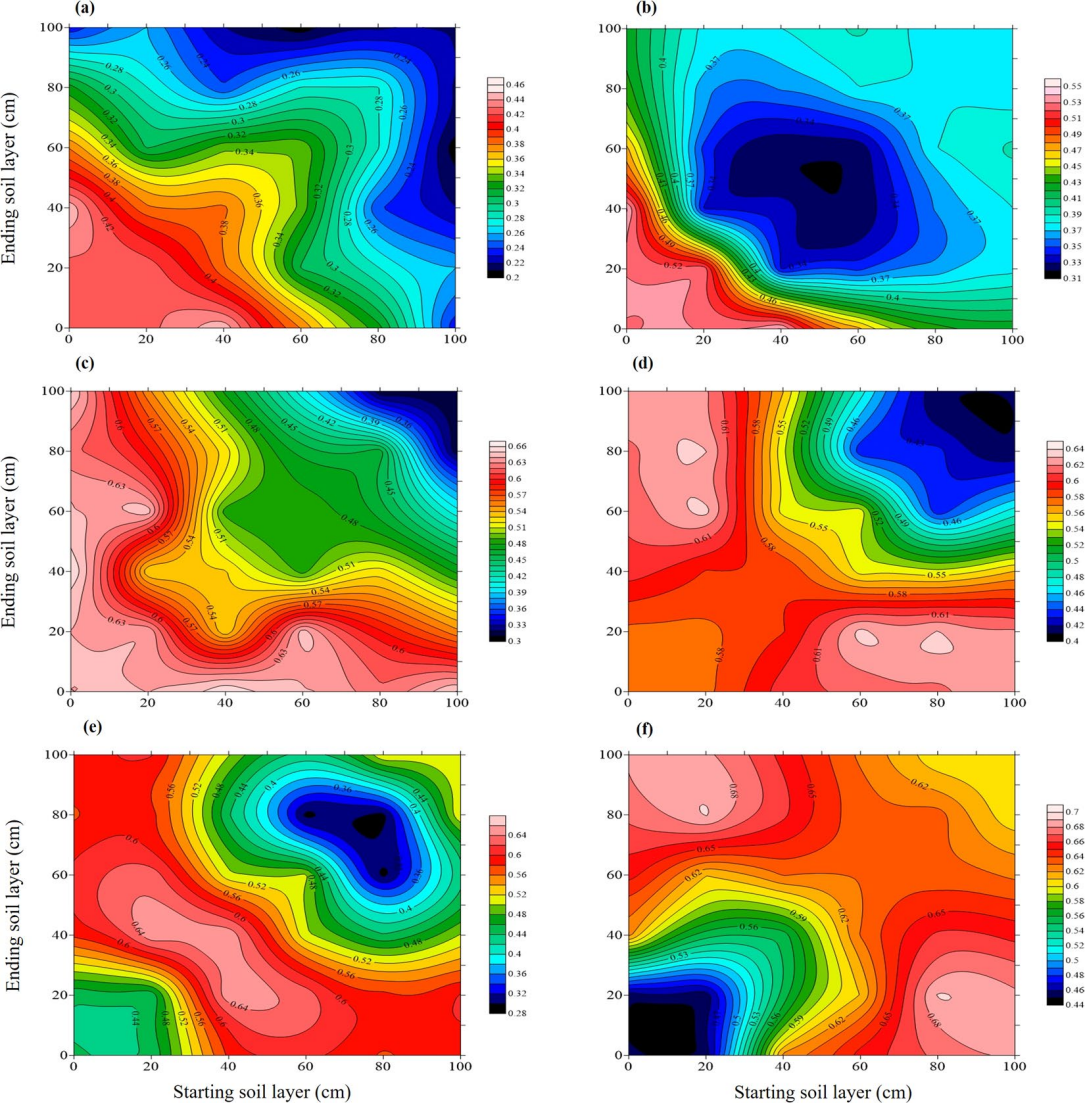
Correlation between increased aboveground N uptake and NO_3_^−^-N in different soil layers. The ‘increased’ denotes the difference between values under each N treatment compared to N0 treatment. (**a–f**) represent datasets at wintering (**a**), reviving (**b**), jointing (**c**), booting (**d**), anthesis (**e**) and maturity (**f**).

The correlation coefficients (r) between increased aboveground N uptake and increased NO_3_^−^-N were plotted in Fig. 4. The results showed a poor correlation at early growth stages (wintering-reviving), with an improvement in performance in mid to later stages. The upper soil layer combinations gave larger *r* amplitude variation than lower during wintering, jointing, booting and anthesis, whereas an opposite trend was observed at reviving and maturity. These inconsistencies suggest that environmental factors such as site, year, temperature and precipitation greatly affect the relationship between increased aboveground N uptake and increased NO_3_^−^-N content. The high *r* region gradually moved down with growth due to the simultaneous downward movement of NO_3_^−^-N and development of the root system, with the most significant *r* region primarily concentrated in the 0–40 cm layer during wintering-reviving, the 20–60 cm layer during jointing-anthesis, and the 20–80 cm layer during maturity.

**Figure 5:**
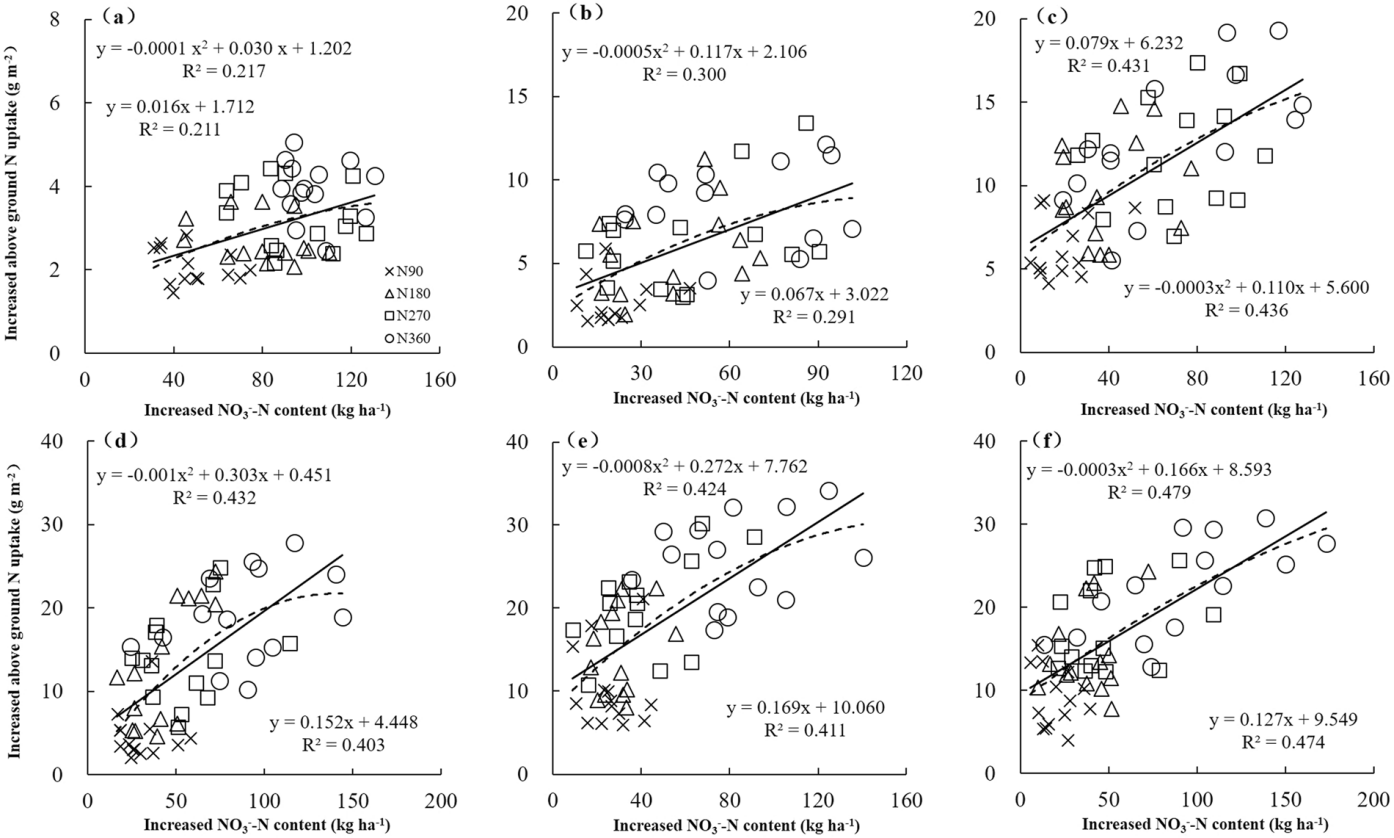
Optimal relationships between increased aboveground N uptake and NO_3_^−^-N at different growth stages (*n* = 60). (**a**) At wintering in the 0–40 cm soil layer, (**b**) reviving in the 0–40 cm soil layer, (**c**) jointing in the 20–60 cm soil layer, (**d**) booting in the 20–60 cm soil layer, (**e**) anthesis in the 20–60 cm soil layer and (**f**) maturity in the 20–80 cm soil layer.

The best performance at different growth stages based on *R*^2^ between increased aboveground N uptake and increased NO_3_^−^-N was subsequently plotted to further understand the relationship between soil N supply and plant N demand (Fig. 5). The greatest *R*^2^ ranged from 0.211 to 0.479 across different growth stages, and was >0.402 after jointing due to strong demand for N. Meanwhile, increased aboveground N uptake throughout the six growth stages was positively correlated with the increase in NO_3_^−^-N, with saturation during booting-maturity. When the N application rate was greater than 270 kg ha^−1^, the soil N supply was far greater than the plant N demand, resulting in accumulation of residual NO_3_^−^-N in the soil.

### Yield, aboveground N uptake, NO_3_^−^-N residues and NUE at harvest

**Figure 6:**
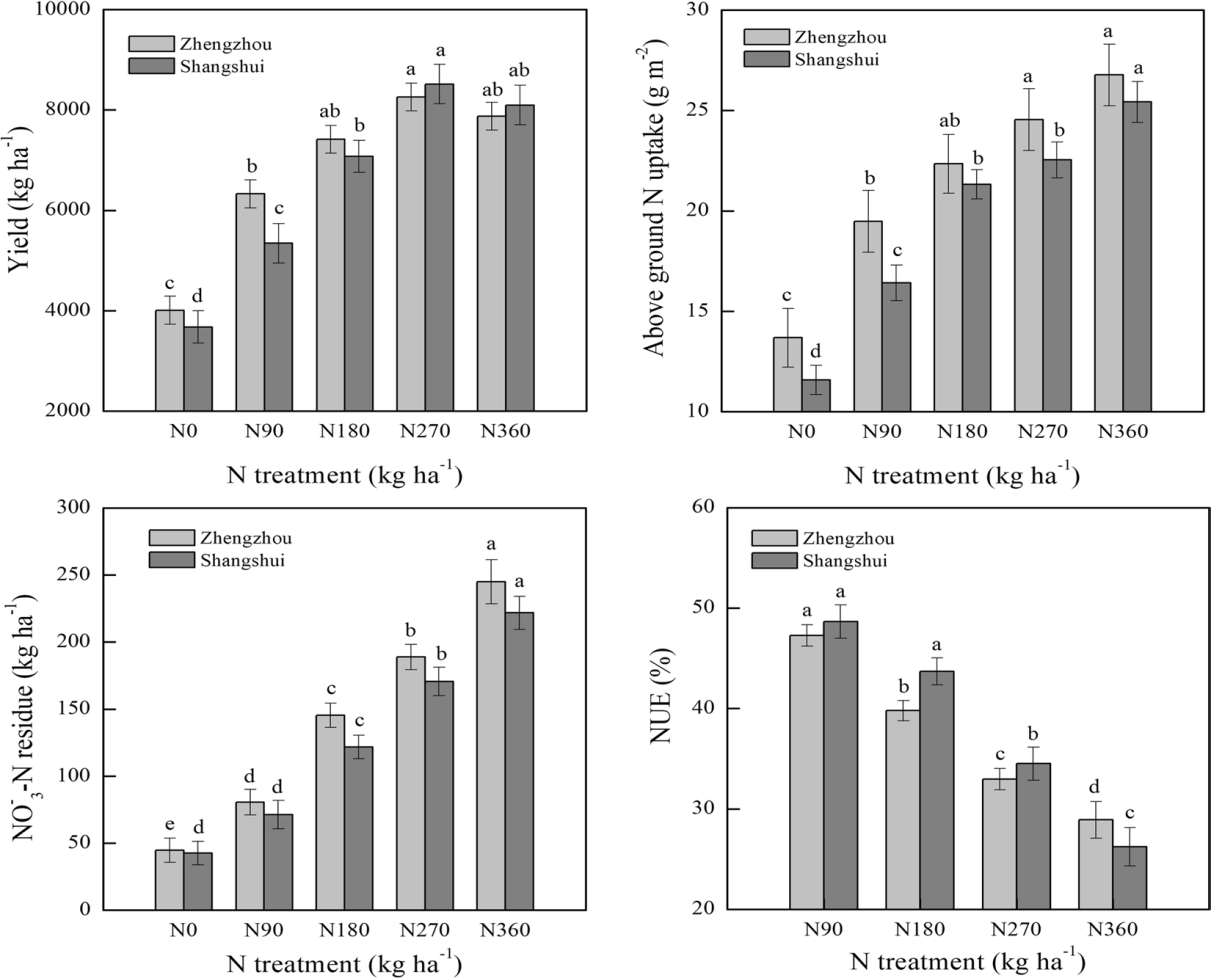
Effect of N rate on yield, aboveground N uptake, NO_3_^−^-N residues and NUE at harvest in each site. Vertical bars represent standard errors and different letters show significance at 0.05.

Winter wheat yield, aboveground N uptake, NO_3_^−^-N residues and NUE varied among N treatments and between sites (Fig. 6). The effect of N application on yield in Zhengzhou was consistent with that in Shangshui, all treatments ranking in the order of N270 > N360 > N180 > N90 > N0, with no significant differences between N270 and N360. Yield was higher in Zhengzhou than Shangshui at N application rates <180 kg ha^−1^, with an opposite trend at >270 kg ha^−1^. A significant difference in NO_3_^−^-N residues was observed among N treatments, manifesting in the trend of N360 > N270 > N180 > N90 > N0. The increase in NO_3_^−^-N residues rose with increasing N application, especially under N270 and N360, resulting in a high rate of NO_3_^−^-N residues in both Zhengzhou (264 kg ha^−1^) and Shangshui (233 kg ha^−1^). NO_3_^−^-N residues were higher in Zhengzhou than Shangshui under all N treatments, while the increase in NO_3_^−^-N residues resulting from net N application was the same in both sites. NUE decreased with increasing N, and there was a significant difference between N180, N270 and N360 in both Zhengzhou and Shangshui. Compared to Zhengzhou, NUE was higher in Shangshui under N90-N270, with an opposite trend under N360 treatment.

At an N application rate of 270 kg ha^−1^, yield reached a maximum value (8377.3 kg ha^−1^), while N360 reduced yield by 3.5% and NUE by 17.5%, with an increase of 31.5% in NO_3_^−^-N residue. Compared to N270, N180 caused a decrease in yield of 14.1% and in NO_3_^−^-N residues of 25.6%, with an increase of 22.4% in NUE. These findings suggest that more NO_3_^−^-N residues remained in the soil, and yield and NUE both decreased when N fertilizer exceeded 270 kg ha^−1^. A recommended N rate of 180–270 kg ha^−1^ could therefore minimize soil NO_3_^−^-N residues while maintaining high yield and NUE.

## Discussion

A number of recent studies have shown that the spatial-temporal distribution of NO_3_^−^-N in the soil profile varies with the crop growth period and N application rate^14,15^. Fan *et al*.^16^ revealed peak NO_3_^−^-N accumulation in the 140 cm layer under long-term abundant N application in wheat. Meanwhile, Miao *et al*.^11^ pointed out that N absorption in wheat depends on the NO_3_^−^-N content in different soil layers at different stages; the 0–20 cm layer at wintering, 0–40 cm layer at reviving and 0–60 cm layer at jointing. In this study, soil NO_3_^−^-N experienced two peaks, a main peak and second peak at wintering and booting, respectively. This was possibly due to base N application at pre-sowing and top dressing of N fertilizer at jointing. In addition, the highest accumulation of NO_3_^−^-N was mainly concentrated in the 0–40 cm layer before anthesis and the 60–80 cm layer at maturity, possibly due to irrigation and high rainfall in mid to late growth stages, which will have promoted downward movement of NO_3_^−^-N. Encouraging the spatial-temporal distribution of NO_3_^−^-N via optimal irrigation and nitrogen management is therefore important in meeting the needs of wheat N absorption.

An optimal N management regime matches the seasonal demand of a crop with the soil supply^17^. Miao *et al*.^11^ suggested that cumulative nitrate N was significantly correlated with wheat seed yield and shoot weight, while Wang *et al*.^18^ revealed that the amount and percentage increase in wheat yield were significantly negatively correlated with soil accumulation of NO_3_^−^-N in the 0–80 and 0–100 cm layers, respectively. In this study, a significant correlation between increased aboveground N uptake and increased NO_3_^−^-N content was observed. Moreover, at an N application rate >270 kg ha^−1^, the relationship was subject to saturation during booting-maturity, suggesting that N rates above 270 kg ha^−1^ exceed wheat N demands. In addition, optimal performance was observed in the upper soil layer (0–40 cm) before anthesis, mainly due to limited extension of the roots during these stages and the high NO_3_^−^-N content in the upper layers. Fertilizer should therefore be mechanically applied to ensure it is better absorbed by the roots from the upper and middle soil layers. After anthesis, the root system grew downward, as did NO_3_^−^-N due to irrigation and rainfall, and the optimal relationship was therefore found in deeper soil layers (60–80 cm). In mid to late growth stages, flood irrigation should therefore be avoided to prevent NO_3_^−^-N from infiltrating too deeply, thereby allowing the root system to fully absorb nutrients in mid to deep soil layers. Further understanding of the relationship between N supply and demand is therefore required to provide a detailed theoretical basis for wheat N fertilizer management.

Roots are important for absorption and metabolism, contributing to the growth of aboveground tissues. Root growth and development varies according to N fertilizer management. In this study, the highest root weight density was observed under low N treatment (90 kg ha^−1^), while under high N application a reduction in root weight density was observed at different growth stages in both Zhengzhou and Shangshu (Fig. 2). These results are in agreement with earlier studies, whereby high N fertilization had a negative effect on root growth^19,20^. The roots were mainly distributed in the upper and middle soil layers (the 0–60 cm layer accounting for more than 80%) (Fig. 2). This may be due to the fact that the early growth stages are accompanied by higher availability of resources (water and nutrients) in the top soil layers, which encourages root proliferation in the upper soil layer^21^. Many studies have shown that distribution of the root system in winter wheat is closely related to crop yield, the water absorption capacity, and nutrient absorption capacity^22,23^. Correlations between aboveground N uptake and root weight density were therefore examined. As a result, aboveground N uptake was found to be positively correlated with the root weight density in the upper to mid soil layers (except at maturity; Table 5). Wang *et al*.^19^ revealed that root development in the 20–60 cm soil layer during jointing was highly beneficial in terms of yield in winter wheat. Moreover, Palta *et al*.^24^ also found that deep root growth in wheat was correlated with water uptake, while Kamiji *et al*.^25^ revealed that the correlation between wheat shoot N content and root biomass was siginificant under low N supply. Root growth and development is therefore affected by the complex belowground environment, and thus, further efforts are required to fully understand the role of the root system in terms of yield.

N fertilizer application was previously found to play a significant role in realizing the maximum potential of crop production and environmental sustainability^26^. A lack of N can affect yield increases, while excess can lead to an increase in soil NO_3_^−^-N accumulation and leaching^5,27^. Wang *et al*.^28^ showed that the optimal N fertilizer rate is around 185 kg ha^−1^ for maximum wheat yield (7000 kg ha^−1^) in the North China Plain. Meanwhile, Dai *et al*.^29^ found that an N application rate of 66–92 kg ha^−1^ reduced losses in N fertilizer and resulted in a relatively high grain yield in winter wheat in the dryland Loess Plateau. In the present study, maximum yield was obtained at 270 kg ha^−1^ N, with a slight decrease at >270 kg ha^−1^, accompanied by a sharp increase in the NO_3_^−^-N content, and significant decrease in NUE. Compared to 270 kg ha^−1^, yield remained high at 180 kg ha^−1^ N (a decrease of 14.1%), and residual NO_3_^−^-N and NUE improved. To balance wheat yield and the risk of environmental pollution in the study area, while ensuring a well-developed root system, an N application rate of 180–270 kg ha^−1^ is therefore recommended. It should be noted, however, that this study was conducted on winter wheat in Henan province only, and thus, the adaptability of the results should be validated with wheat grown in other environments.

## Conclusions

Balancing the relationship between high yield and environmental sustainability is important for agricultural development. To understand how to maximize yield and minimize resource waste, field experiments were conducted to explore the balance between N supply and demand in winter wheat. Increased aboveground N uptake was positively correlated with increased NO_3_^−^-N until a plateau response at an N rate >270 kg ha^−1^, with optimal relationships observed in the 20–60 cm layer during jointing-anthesis and the 20–80 cm layer at maturity. The root weight density in the 20–60 cm layer accounted for 30% of the total root system, and these roots played a vital role in the absorption and transportation of nutrients. Meanwhile, a maximum root weight density was observed at 90 kg ha^−1^ N, suggesting that a reduction in N fertilizer input is beneficial to root development and distribution. In the experimental area, economic benefits increased and environmental risk decreased at an N rate of 180–270 kg ha^−1^ under sufficient irrigation, when comprehensively taking yield, NUE and residual NO_3_^−^-N into account.

## Materials and Methods

### Experimental design

Different N fertilizer rates were examined across three years at two sites in Henan Province, China: Zhengzhou city in the growing seasons of 2013–2016 and Shangshui city in the seasons of 2014–2016. The fluvo-aquic soil in Zhengzhou consisted of 16.17–17.35 g kg^−1^ organic matter, 0.83–0.94 g kg^−1^ total N, 97.25–104.20 mg kg^−1^ available N, 16.25–18.85 mg kg^−1^ available P, and 198.45–245.63 mg kg^−1^ available K. In Shangshui, the lime concretion black soil consisted of 19.59–20.36 g kg^−1^ organic matter, 1.19–1.47 g kg^−1^ total N, 85.33–97.25 mg kg^−1^ available N, 6.65–9.74 mg kg^−1^ available P, and 166.23–195.35 mg kg^−1^ available K. The cultivar in all experiments was Yumai 49–198.

N treatment was carried out at a rate of 0, 90, 180, 270 and 360 kg ha^−1^. N fertilizer was applied as urea using 50% of the N supply prior to seeding and the final 50% at the jointing stage. Prior to seeding, 150 kg ha^−1^ P_2_O_5_ (as monocalcium phosphate [Ca(H_2_PO_4_)_2_]) and 90 kg ha^−1^ K_2_O (as KCl) were also applied in all treatments. The daily mean temperature was 16–19 and 12–15 °C at base and topdressing fertilizer application, respectively. The experimental field in Zhengzhou was irrigated at jointing and flowering, while in Shangshui irrigation was carried out at jointing. Irrigation was applied at a rate of 750 m^3^ ha^−1^. Average precipitation in the mid to late growth stage(April 1 to June 1) was 90–120 mm. A randomized complete block design was used for all experiments with three replicates each. The plot size was 7 × 2.9 m in Zhengzhou city and 6 × 4.5 m in Shangshui city. Plant and soil samples were obtained at wintering, reviving, jointing, booting, anthesis and maturity. All other management followed local standard practices of wheat production. Herbicides and pesticides were used to control weeds and pests.

### Aboveground N uptake analysis

Aboveground wheat samples were obtained from three areas of 0.2 m^2^ in each plot and used to determine the aboveground dry weight and N concentration. All samples were oven-dried at 70 °C until they reached a constant weight. Dried ground samples were then passed through a 1 mm screen and stored in plastic bags for chemical analysis. The aboveground N content was determined using the micro-Kjeldahl method. N uptake was estimated by multiplying the aboveground dry matter by the N concentration.

### N use efficiency analysis

NUE was determined based on the N uptake and N fertilizer application rate as follows:

1 $$NUE( \% )=\frac{{U}_{N}-{U}_{0}}{{F}_{N}}\times 10\times 100 \% $$

where U_N_ is the total N uptake (gm^−2^) under fertilizer treatment and U_0_ is N uptake (g m^−2^) under no-fertilizer treatment. F_N_ represents the rate of N fertilizer application (kg ha^−1^), and 10 is the conversion coefficient.

### Yield analysis

At maturity, spike numbers at fixed sampling points of 1 m long in a double row were investigated for each treatment. Twenty plants were selected randomly for measurements of spike number, and two samples of 500 grains each were counted and weighed to determine the thousand grain weight. All plants within an area of 4 m^2^ (4 × 1 m^2^ in Zhengzhou and 2 × 2 m^2^ in Shangshui) were selected randomly in each plot and harvested manually close to the ground. After air drying, the samples were threshed and weighed to determine the grain yield.

### Soil nitrate-N analysis

Two core soil samples were collected from the 0–20, 20–40, 40–60, 60–80 and 80–100 cm soil layers using an auger (inner diameter: 4.0 cm) at wheat sowing, wintering, reviving, jointing, booting, anthesis and maturity, respectively. Five soil cores were sampled at each plot, and soil samples from the same layer in the same plot were mixed and stored in a labeled plastic bag. Fresh soil samples weighing 5.0 g were extracted with 50 mL of 1 mol L^−1^ KCl by shaking for 1 h, and the NO_3_^−^-N concentration in the filtrate determined immediately using a continuous flow analyzer (AA3). The soil NO_3_^−^-N concentration was then expressed based on the oven-dried soil. NO_3_^−^-N in the 0–100 cm soil layer was calculated as follows according to Dai *et al*.^29^:

2 $${\rm{A}}=\frac{{C}_{{\rm{i}}}\times {D}_{i}\times {T}_{i}}{10}$$

where A is soil NO_3_^−^-N accumulation (kg ha^−1^), C_*i*_ is the soil NO_3_^−^-N concentration (mg kg^−1^), D_*i*_ is the soil bulk density (g cm^−3^), T_*i*_ is the soil layer thickness (cm), *i* = 20, 40, 60, 80 and 100, and 10 is the conversion coefficient.

### Root sampling

Roots were sampled using an auger (inner diameter: 10.0 cm, length: 20.0 cm) at wintering, reviving, jointing, booting, anthesis and maturity in 2014–2015 in both sites. Three sampling points were randomly selected in the fixed sampling zone of each plot as follows: (l) within the row, (2) between rows, and (3) in an intermediate position, i.e. with one edge of the core touching the row^30^. At each sampling point, drilling was carried out three times. Samples of three drillings were combined as one soil sample. Intervals of 20 cm were used to represent different soil layers up to a depth of 1 m. Each sample was placed in a 100-mesh nylon bag and washed with tap water to remove impurities from the root samples. The samples were then oven-dried at 80 °C to a constant weight and weighed. Root weight density (RWD) refers to the root dry weight in one unit volume of soil and was calculated as follows:

3 $$RWD=\frac{M}{V}\times {10}^{6}$$

where RWD is the root weight density (g m^−3^), M is the root dry weight (g), and V is the volume of the soil sample (cm^3^).

### Data analyses

Wheat yield, NUE and soil NO3^−^-N content were analysed and the experimental effects were assessed using analysis of variance (ANOVA) with SPSS version 19.0. The means of each treatment were also compared using the least significant difference (LSD) test at a probability level of p ≤ 0.05. The NO_3_^−^-N content and relationships between aboveground N uptake and NO_3_^−^-N content were analyzed using Surfer 10.0.

## References

[1] Organization A (2012). FAO Statistical Pocketbook 2012: world food and Agriculture. FAO Statistical Pocketbook World Food & Agriculture.

[2] Ju XT (2009). Reducing environmental risk by improving N management in intensive Chinese agricultural systems. Proc. Natl. Acad. Sci. USA.

[3] Hatfield JL, Gitelson AA, Schepers JS, Walthall CL, Pearson CH (2008). Application of spectral remote sensing for agronomic decisions. Agronomy Journal.

[4] Miao Y, Stewart BA, Zhang F (2011). Long-term experiments for sustainable nutrient management in China. A review. Agronomy for Sustainable Development.

[5] Ju XT, Liu XJ, Zhang FS (2003). Accumulation and movement of NO_3_^−^-N in soil profile in winter wheat-summer maize rotation system. Acta Pedologica Sinica.

[6] Lenka S, Singh AK, Lenka NK (2013). Soil water and nitrogen interaction effect on residual soil nitrate and crop nitrogen recovery under maize–wheat cropping system in the semi-arid region of northern India. Agriculture Ecosystems & Environment.

[7] Zheng CY, Yu ZW, Wang D, Zhang YL, Shi Y (2012). Effects of tillage practices on nitrogen accumulation and translocation in winter wheat and NO_3_^−^-N content in soil. Plant Nutrition and Fertilizer Science.

[8] Feng B (2012). Effect of nitrogen application level on nitrogen use efficiency in wheat and soil nitrate-N content under bed planting condition. Acta Agronomica Sinica.

[9] Xia XL (2010). Effects of nitrogen fertilization on spatial-temporal distributions of soil nitrate and nitrogen utilization in wheat season of rice-wheat systems. Acta Pedologica Sinica.

[10] Wang XK, Li ZB, Xing YY (2015). Effects of mulching and nitrogen on soil temperature, water content, nitrate-N content and maize yield in the Loess Plateau of China. Agric. Water Manage.

[11] Miao YF, Wang ZH, Li SX (2015). Relation of nitrate N accumulation in dryland soil with wheat response to N fertilizer. Field Crops Research.

[12] Cui ZL (2007). Appropriate soil nitrate N content for a winter wheat/summer maize rotation system in North China Plain. Chinese Journal of Applied Ecology.

[13] Cui ZL (2008). Soil nitrate-N levels required for high yield maize production in the North China Plain. Nutrient Cycling in Agroecosystems.

[14] Malhi SS (2003). Light fraction organic N, ammonium, nitrate and total N in a thin Black Chernozemic soil under bromegrass after 27 annual applications of different N rates. Nutr. Cycl. Agroecosys.

[15] Elbasiouny H, Abowaly M, Abu-Alkheir A, Gad AA (2014). Spatial variation of soil carbon and nitrogen pools by using ordinary Kriging method in an area of north Nile Delta, Egypt. Catena.

[16] Fan J, Hao MD, Dang JH (2000). Distribution and Accumulation of NO_3_^−^-N in Soil Profile of Long-term Located Fertilizer Experiment. Soil & Environmental Sciences.

[17] Thiyagarajan TM, Stalin P, Dobermann A, Cassman KG, Berge HFMT (1997). Soil N supply and plant N uptake by irrigated rice in Tamil Nadu. Field Crops Research.

[18] Wang ZH, Miao YF, Li SX (2015). Effect of ammonium and nitrate nitrogen fertilizers on wheat yield in relation to accumulated nitrate at different depths of soil in drylands of China. Field Crops Research.

[19] Wang CY (2014). Effects of different irrigation and nitrogen regimes on root growth and its correlation with above-ground plant parts in high-yielding wheat under field conditions. Field Crops Research.

[20] Elazab A, Serret MD, Araus JL (2016). Interactive effect of water and nitrogen regimes on plant growth, root traits and water status of old and modern durum wheat genotypes. Planta.

[21] King J (2003). Modelling Cereal Root Systems for Water and Nitrogen Capture: Towards an Economic Optimum. Ann Bot.

[22] Chen XY, Liu XY, Luo YP (2003). Effects of soil moisture on dynamic distribution of dry matter in winter wheat root and shoot. Scientia Agricultura Sinica.

[23] Zhang X, Chen S, Sun H, Wang Y, Shao L (2009). Root size, distribution and soil water depletion as affected by cultivars and environmental factors. Field Crops Research.

[24] Palta JA (2011). Large root systems: are they useful in adapting wheat to dry environments?. Functional Plant Biology.

[25] Kamiji Y, Pang J, Milroy SP, Palta JA (2014). Shoot biomass in wheat is the driver for nitrogen uptake under low nitrogen supply, but not under high nitrogen supply. Field Crops Research.

[26] Li SX, Wang ZH, Hu TT, Gao YJ, Stewart BA (2009). Nitrogen in dryland soils of China and its management. Advances in Agronomy.

[27] Ju X, Liu X, Zhang F, Roelcke M (2004). Nitrogen fertilization, soil nitrate accumulation, and policy recommendations in several agricultural regions of China. Ambio.

[28] Wang H (2017). An optimal regional nitrogen application threshold for wheat in theNorth China Plain considering yield and environmental effects. Field Crops Research.

[29] Dai J (2015). Optimizing nitrogen input by balancing winter wheat yield and residual nitrate in soil in a long-term dryland field experiment in the Loess Plateau of China. Field Crops Research.

[30] Bolinder MA, Angers DA, Dubuc JP (1997). Estimating shoot to root ratios and annual carbon inputs in soils for cereal crops. Agriculture Ecosystems & Environment.

